# A 10-Year Study of Neonatal Sepsis from Tuen Mun Hospital, Hong Kong

**DOI:** 10.3390/pathogens14030276

**Published:** 2025-03-13

**Authors:** Pascoe Lee, Eugene Sin, Kam-Tong Yip, Kenneth Ng

**Affiliations:** Department of Clinical Pathology, Tuen Mun Hospital, Hospital Authority, Hong Kong, China

**Keywords:** anti-infective agents, candidiasis, invasive, drug resistance, microbial, microbial sensitivity tests, neonatal sepsis

## Abstract

Background: Neonatal sepsis is a major cause of infant mortality, and it accounts for a significant consumption of antimicrobials in paediatrics. This is the first comprehensive study on neonatal sepsis in Hong Kong. Methods: From 2014 to 2023, all neonates admitted to a single institution with culture-proven infections from the blood and/or cerebrospinal fluid were selected and reviewed retrospectively. The infecting organisms, their antibiotic nonsusceptibility pattern, and the concordance of empirical antimicrobial therapy with the microbiological profiles were described and were further compared between infants of normal/low birth weight (≥1.5 kg) and very low/extremely low birth weight (<1.5 kg), early-onset sepsis (<72 h), and late-onset sepsis (4–28 days), the first and the second 5-year periods (2014–2018 vs. 2019–2023). Results: After contaminants were excluded, there were 118 affected neonates with 125 organisms identified. Fifty-nine were male. Thirty-four were very low/extremely low birth weight infants, and twenty-eight infants had early-onset sepsis. Patient demographics and the microbiology findings did not differ between the first 5 years and the latter 5 years. However, the incidence of neonatal sepsis was significantly lower in the latter 5 years (3.23 vs. 1.61 per 1000 live births, *p* < 0.001), the period that coincided with the COVID-19 pandemic. *Escherichia coli* was the most common Gram-negative pathogen. *Streptococcus agalactiae* and *Streptococcus bovis* group infections were more common in early-onset sepsis, while coagulase-negative *Staphylococcus* and non-*E. coli* Gram-negative pathogens were more likely to occur in late-onset sepsis. In very low/extremely low birth weight infants, the rate of cefotaxime or ceftriaxone nonsusceptibility among Gram-negative isolates was higher (*p* = 0.01), and concordance of empirical antimicrobial therapy was lower (*p* = 0.006). Conclusions: Management of neonatal sepsis remains challenging, and there is a need for optimising antimicrobial therapy, especially in preterm patients. Antepartum screening with intrapartum antibiotic prophylaxis is effective in reducing the risk of early-onset sepsis associated with *S. agalactiae*, while stringent infection control measures are important for the prevention of late-onset sepsis.

## 1. Introduction

Neonatal sepsis, invasive bacterial and fungal infections affecting newborn infants within the first 28 days of life, is a significant cause of neonatal morbidity and mortality. Neonatal sepsis is universal, but its epidemiology is often shaped by socioeconomic and biological factors. The incidence is higher in low- and middle-income countries [1,2]. Compared to term neonates, preterm neonates are especially susceptible to sepsis and its associated mortality [3]. Over the last two decades, antenatal screening and intrapartum antibiotic prophylaxis against group B streptococcal infection have been practised in many parts of the world [4,5] and are now recommended by the World Health Organization [6]. Nevertheless, neonatal sepsis remains a significant disease burden, and it is still increasing in some parts of the world [1,7].

The diagnosis and treatment of neonatal sepsis is challenging. Clinical manifestations of neonatal sepsis are often non-specific [8,9], so positive cultures of specimens from normally sterile sites are recognised as the gold standard for the diagnosis [10,11]. However, the sensitivity of such cultures is limited by the often low-volume specimens sampled from neonates [12], and antimicrobial consumption is high in both culture-negative and culture-positive cases [13]. A combination of an aminoglycoside and a penicillin antibiotic has become a widespread choice of empiric therapy for neonatal sepsis for nearly half a century [14] and continues to be the recommended regimen from prominent bodies [15,16,17] despite the evolving aetiology and antimicrobial resistances, especially in lower-income regions [18].

Neonatal sepsis is conventionally classified into early-onset sepsis and late-onset sepsis, of which the latter has a higher case load [19]. Infections that occur in the first 72 h of life can often be traced to perinatal transmission. Those that occur beyond the first 3 days of life have a wide variety of aetiologies, including nosocomial transmission. The microbiological profiles, including pathogens and antibiotic susceptibility, are thus different between the two kinds of neonatal sepsis. Continuous surveillance, both at the institutional and national levels, is important to track the evolving epidemiology and thus to guide effective clinical management. With the recent implementation of universal antenatal screening for maternal group B *Streptococcus* colonisation and intrapartum antibiotic prophylaxis, there is a growing interest to study early-onset neonatal sepsis, especially neonatal group B streptococcal infection in Hong Kong. As there has not been any systematic study about neonatal sepsis in Hong Kong, we set out to understand the nature of this condition, including the microbiological profile and susceptibility of the microbial isolates, the kinds of neonates affected in terms of birth weight, the differences between early- and late-onset infections, and patterns of empirical antimicrobial treatments, by examining it through one of the major regional neonatal units in the city over a 10-year period.

## 2. Materials and Methods

This is a retrospective study of clinical and laboratory data on patients with neonatal sepsis admitted to Tuen Mun Hospital. This is one of the major hospitals in Hong Kong that serves 17.1% of the total population by the year 2021 [20]. The study period was from January 2014 to December 2023, inclusively. Sepsis was defined by the presence of pathogen(s) in the blood and/or cerebrospinal fluid (CSF) identified by the laboratory via culture.

Blood samples were collected in BACTEC Peds Plus/F culture vials (Becton Dickinson, Sparks, MD, USA). The vials were incubated in the BD BACTEC FX blood culture system (Becton Dickinson, Sparks, MD, USA) for up to 5 days, and vials flagged positive were further subcultured onto solid culture media. CSF samples were collected in sterile glass Bijou bottles and inoculated onto solid culture media and Thiol broth (Sigma-Aldrich, St. Louis, MO, USA) enriched with pyridoxal hydrochloride (Sigma-Aldrich, St. Louis, MO, USA) and Vitox supplement (Oxoid Ltd., Basingstoke, UK) for up to 3 and 5 days, respectively. Growth on culture media was isolated and identified using the available methods (including conventional methods, API identification kits (bioMérieux, Marcy-l’Étoile, France), and BD Phoenix (Becton Dickinson, Sparks, MD, USA) automated identification system, and, since August 2016, the Bruker MALDI Biotyper system (Bruker Daltonik GmbH, Bremen, Germany) as well). Antimicrobial susceptibility testing and interpretation were performed according to Clinical and Laboratory Standards Institute guidelines.

Subjects were included when they were under the chronological age of 28 days of life at the time of microbiological sampling. *Bacillus*, *Corynebacterium*, *Cutibacterium*, *Lactobacillus*, and *Micrococcus* spp. were considered culture contaminants [21,22] in the present study and were excluded from analysis. For coagulase-negative *Staphylococcus* (CoNS), the cultures were considered significant if they were treated with at least 5 days of effective antibiotics [23,24] with reference to susceptibility results; otherwise, they were also classified as contaminants and were excluded. If a patient has more than one culture request growing the same organism within the period of antibiotic treatment, those positive culture requests would count towards a single organism.

Patient demographics, including the age at the first microbiological culture request that turned positive, gestation in weeks, birth weight in kilograms, and mortality, defined as death within 7 days of a positive blood/CSF culture, were recorded. Additional information collected includes whether patients, prior to the sepsis episode, were ever discharged into the community (defined as out-born) or had been hospitalised since birth (defined as in-born), any systemic antibiotic given in the intrapartum or neonatal period, as well as the antimicrobial choices used.

The study period was divided into two five-year periods, the first five years 2014 to 2018, and the second five years 2019 to 2023. Patients were divided into two groups according to birth weight, the very low/extremely low birth weight (V/ELBW, <1.5 kg) and the normal/low birth weight (N/LBW, ≥1.5 kg) groups. The infections were classified into early-onset sepsis (EOS) if the positive cultures were obtained when the patient was under the age of 72 h of life and into late-onset sepsis (LOS) if patients were older than 72 h of life at the time of specimen collection [9,16,21]. LOS was further classified into healthcare-associated (HA-LOS) if the positive cultures were obtained more than 48 h after admission, including patients who were discharged for less than 48 h before readmission, and community-acquired (CA-LOS) otherwise [25,26,27].

Empiric therapy was defined as antibiotic treatment, alone as monotherapy or in combination therapy, used after blood and/or CSF samples were taken and prior to the report of positive microbiological findings. Empiric therapy was classified as concordant if at least one of the empirical antimicrobials being used was considered an agent for which the pathogen was susceptible based on identification and susceptibility testing, and as discordant if the pathogen was not susceptible to any of the empiric antimicrobials used for that patient.

The included patients’ demographic data, microbiological findings, and treatment relevance were described. Mortality cases would be presented. The demographic data and microbiological findings were compared between the first and the second five-year periods to see if there were any changes over time. Microbiological data, including antibiotic nonsusceptibility, were compared between the different birth weight groups, EOS versus LOS, and between CA-LOS and HA-LOS.

Statistical analysis was performed with Stata 14.2 (StataCorp, College Station, TX, USA). Odds ratios (ORs) with 95% confidence intervals (95% CIs) would be computed. Non-parametric data were compared by Fisher’s exact test. A *p*-value < 0.05 was considered significant.

## 3. Results

A total of 161 neonates were identified with microorganisms found in the blood and/or CSF cultures during the study period. Among them, 43 were excluded as the pathogens were considered contaminants. The remaining 118 neonates formed the subjects of this study, and their demographics are listed in Table 1. The patients’ demographic data were comparable between the two periods.

The total number of live births during the 10-year study period was 45,555, with 27,552 from 2014 to 2018 and 18,003 from 2019 to 2024 [28]. Thus, neonatal sepsis occurred at an annual incidence of 2.59 per 1000 live births. Comparing the figures between the first and second 5-year periods, there was a significant drop in the incidence of neonatal sepsis in the latter period (3.23 vs. 1.61 per 1000 live births, respectively; *p* < 0.001). When it was analysed separately, the difference was not observed among infants with early-onset neonatal sepsis (0.76 vs. 0.38 per 1000 live births; *p* = 0.13). However, the incidence of late-onset neonatal sepsis was significantly lower in the latter 5-year period (2.52 vs. 1.22 per 1000 live births; *p* = 0.003). This latter period from 2019 to 2024 coincided with the global COVID-19 pandemic.

Of the 118 neonates, a total of 125 organisms were found. Among them, 117 organisms were identified from the blood only, 2 from CSF only, and 6 were positive in both the blood and the CSF. From the patient perspective, 113 had a single pathogen, 3 were infected by two organisms, and 2 had three organisms. The list of pathogens is listed in Table 2. Comparing the first and the second 5-year periods, the distribution of Gram-positive bacteria, Gram-negative bacteria, and fungi did not differ.

The pattern of infection was compared between N/LBW and V/ELBW infants (Table 3). The V/ELBW infants were more often exposed to prior antibiotic treatment, and they had more infections with CoNS and yeasts.

When EOS and LOS were compared (Table 4), streptococcal infections were more common in EOS, especially *Streptococcus agalactiae* (group B *Streptococcus*, GBS) and *Streptococcus bovis* group (SBG). On the other hand, CoNS and Gram-negative bacteria other than *Escherichia coli* were more commonly seen in LOS. Among those who presented with CA-LOS, the affected were exclusively N/LBW infants. GBS was exclusively encountered among CA-LOS cases (Table 5).

Eight (6.8%) neonates died. Their clinical and laboratory features are listed in Table 6. All neonates were premature, and seven of them were born at 25 weeks of gestation or less. The pathogens included four yeasts, three Gram-negative bacteria, and one GBS.

Concerning antibiotic nonsusceptibility of the bacterial pathogens (Table 7), most of the staphylococcal isolates were methicillin-resistant. This was even more significant among infants of V/ELBW. On the other hand, the nonsusceptibility of streptococcal isolates to penicillin was uncommon, with only two *Streptococcus mitis* group isolates being nonsusceptible. All *Enterococcus faecalis* strains were ampicillin-susceptible. No vancomycin resistance was found among the Gram-positive isolates. The majority of the Gram-negative pathogens were nonsusceptible to ampicillin, while nonsusceptibility to gentamicin and cefotaxime or ceftriaxone varied, with organisms found from the V/ELBW group exhibiting a higher rate of cefotaxime or ceftriaxone nonsusceptibility. No carbapenem resistance was found among the Gram-negative isolates.

The vast majority of patients, 112 (94.9%) out of 118, were empirically prescribed at least two antimicrobials. Monotherapy was used empirically in four cases. One patient did not receive empirical antimicrobials prior to the positive culture result, and another died before the commencement of empirical antimicrobials.

Throughout the study period, both ampicillin and cefotaxime were the most commonly prescribed antibiotics, and the combination corresponded to the most popular regimen used in the neonatal unit (Table 8). However, cefotaxime was used more often in the second 5-year period, while the usage of gentamicin went out of favour. On the other hand, there was a reciprocal relationship between the use of ampicillin and vancomycin when infants with different birth weights were compared. Ampicillin was preferred in N/LBW infants while vancomycin was used more often in V/ELBW infants. Among infants with LOS, the combination of cefotaxime with either ampicillin or vancomycin appeared to be the most popular regimen.

Concordance of empirical antimicrobial therapy and antibiotic susceptibility was achieved in more than 60% of cases (Table 9). It appeared to be lower during the second 5-year period and in late-onset sepsis, but the difference was not statistically significant. However, the concordance rate was significantly poorer in septic V/ELBW infants.

## 4. Discussion

The current study provides the first overview of the microbiological profile of neonatal sepsis, both early- and late-onset sepsis, in Hong Kong over a period of 10 years. Although the number of infected neonates was fewer during the last five years as compared with the first, likely because of the COVID-19 pandemic [29], patient demographics such as the proportion of V/ELBW infants, the ratio of inborn to out-born infants, and the proportion of newborns receiving intrapartum antibiotics prior to the onset of sepsis remained the same. Also, the proportions of the Gram-positive bacteria, Gram-negative bacteria, fungi, the distribution of common pathogens, and the rate of antibiotic resistances were comparable between the first and the second 5-year periods.

The sharp change in the rate of neonatal sepsis between the first 5 years (2014–2018) and the second 5 years (2019–2023) was an unexpected finding. This difference was not significant among the cases of EOS but was evident among the cases of LOS. This is understandable as EOS results from in utero transmission or spread of pathogens to the foetus through the maternal genital tract with organisms that commonly colonise the genitourinary and lower gastrointestinal tracts [30]. The COVID-19 pandemic probably did not have any effects on such predisposition. On the other hand, LOS is more often related to nosocomial pathogen transmission and medical interventions during hospitalisation [31]. The fall in the incidence of neonatal sepsis, in particular, HA-LOS, was most likely the result of more stringent isolation, reinforced visitor policies on infection control, and hygienic measures targeted at the facilities for pathogen spread [32,33]. A similar fall in the incidence of LOS during the COVID-19 pandemic has also been observed in India [34], Italy [35], and Sweden [36], but not in the USA [22]. This observation reinforces the importance of infection control measures in neonatal healthcare.

GBS remains one of the major pathogens in neonatal sepsis. In Hong Kong, universal prenatal maternal carriage screening was implemented in 2012, and intrapartum antibiotic treatment against GBS was used in those women who were tested positive [37]. The 16 cases represented an annual incidence of 0.36 per 1000 live births, comparable to the incidence rates of 0.26 and 0.24 per 1000 live births limited to the first seven days of life reported by Chan et al. [38] and Ma et al. [39], respectively. GBS occurred more frequently in the first three days of life, accounting for nearly one-third of the pathogens identified. The infection occurred more often in neonates without intrapartum antibiotic prophylaxis as compared to those who had received antibiotics during the intrapartum or neonatal period. Additionally, in LOS, GBS was exclusively a community-acquired pathogen (*p* < 0.001). The current study was not designed to look into the reasons why newborns continue to be affected by EOS due to GBS in the era of universal antenatal screening, but the unavailability of a timely screening as a result of unplanned or premature delivery and the limited sensitivity of the screening test have been suggested [38]. Nevertheless, GBS remains susceptible to penicillin and is therefore always covered by the current empirical therapy regimens containing ampicillin or cefotaxime.

SBG was another significant Gram-positive pathogen group in neonatal EOS. Apparently, neonatal SBG infection has been rising following the practice of universal antenatal screening and intrapartum antibiotic targeting GBS [37]. Although our strains remained penicillin-susceptible, clinicians should be aware that SBG with reduced susceptibility to penicillin had been uncommonly reported [40,41,42].

CoNS was the most frequent pathogen of LOS, of which the majority of the strains were methicillin-resistant. Because CoNS are skin commensals [43], neonatal CoNS infections are usually considered to be nosocomial in nature, facilitated by biofilm formation in medical devices such as venous catheters [44,45]. Our data showed that neonates in the V/ELBW group were particularly vulnerable to CoNS infections. Although most CoNS species are understood to be of low virulence, premature newborns are at higher risk of CoNS infections than their term counterparts because they often require prolonged hospitalisation and medical interventions [46].

Gram-negative infections occurred in both EOS and LOS. *Escherichia coli* was the only Gram-negative pathogen in EOS, which was consistent with the observation that *Escherichia coli* is the predominant aerobic Gram-negative organism of the birth canal [47]. Non-*Escherichia coli* Gram-negative infections were exclusively seen in LOS in our series. This is similar to the findings from Yale University School of Medicine looking into the microbiology of neonatal sepsis from 1989 to 2003, where the majority of non-*Escherichia coli* Gram-negative infections occurred in neonates aged 5 days or above (5–30 days: 63/90 vs. 0–4 days: 9/35) [48]. A more recent study from Sweden also showed non-*Escherichia coli* Gram-negative infections occurring in LOS more frequently than in EOS (LOS: 51/74 vs. EOS: 9/33) [49]. Our study shows CA-LOS can include organisms that typically affect the older infant, such as *Campylobacter jejuni* and *Neisseria meningitidis*. On the other hand, the neonate with prolonged hospitalisation is susceptible to infections due to Gram-negative organisms, often contributed by suboptimal infection prevention and control practises [50] leading to horizontal transmission by healthcare personnel. In addition, the gut of the preterm neonate is colonised with firmicutes and proteobacteria in greater quantities (as most neonatal aerobic Gram-negative pathogens belong to the latter group) [51,52], often contributed by the environment of the neonatal unit [53] and antibiotic exposures [54]. Gut dysbiosis often precedes LOS [52] and is associated with gut translocation [55,56,57], with studies showing similarity of bacterial strains of bloodstream infections to strains found in the stools of such patients before the onset of infection in neonatal units [58].

The mortality of invasive fungal infection in neonates is particularly high [59,60]. From our data, even among HA-LOS patients in the V/ELBW group, invasive fungal infection was still significantly associated with mortality compared to sepsis due to bacterial organisms (4/7 vs. 3/26, *p* = 0.009). First-line empirical treatment against suspected neonatal invasive candidiasis involves the use of amphotericin B deoxycholate [61,62], but there is a paucity of guidance regarding which neonates should be initiated on empirical antifungal treatment before microbiological results are available. Amphotericin B is not frequently prescribed, as neonatal invasive infection remains uncommon, and amphotericin B is associated with many potential toxicities [63]. As retrospective studies have suggested a possible benefit of early administration of empirical antifungal in cadidaemic neonates [64,65], it would be important to develop recommendations with regard to which patients deserve empirical therapy. On the other hand, guidelines have suggested the use of antifungal prophylaxis in the neonatal unit under specific circumstances [16,61,62], with studies demonstrating variable successes [66,67].

Antimicrobial resistance has emerged as a global problem, and neonatal units are not spared [68]. Poorer survival has been noted in neonates infected with multidrug-resistant organisms, especially carbapenem-resistant Gram-negative organisms [69,70]. In the current study, carbapenem resistance was not an issue, but almost half of the Gram-negative isolates among E/VLBW neonates were nonsusceptible to cefotaxime or ceftriaxone. Although the inclusion of a third-generation cephalosporin as part of the empirical antimicrobial use in neonatal sepsis is an attractive option, such use is associated with an increase in the risk of multidrug-resistant Gram-negative bacterial infection [71] and, especially in preterm infants, candidal colonisation and invasive fungal infections [72,73,74]. A Netherlands study has shown that a regimen containing cefotaxime has a high tendency to result in more cephalosporin resistance in two neonatal units, necessitating the use of meropenem in some patients [75]. Currently, cefotaxime is recommended by guidelines for the empirical treatment of neonatal meningitis, community-acquired neonatal sepsis, and in neonates with microbiological evidence of Gram-negative infections [15,16].

The current study faces several limitations. Although the neonatal unit provided hospitalisation service for all neonates in need in the region served by Tuen Mun Hospital, the single-centre nature of the study limits its generalisability. We have not included patients with culture-negative neonatal sepsis because differentiating from non-infective aetiologies could be difficult [76]. We have not included positive cultures other than blood and CSF, as these are the most common normally sterile specimen types from the neonatal unit. We acknowledge that, in this study, the exclusion of the organisms defined as contaminants, although very rarely causing infections in neonates, may lead to an underestimation of patients with neonatal sepsis, while the approach of using treatment duration for determining the significance of CoNS may lead to overestimation.

## 5. Conclusions

We presented the first epidemiology and microbiology of culture-proven neonatal sepsis from a single institution in Hong Kong in the post-universal antenatal group B *Streptococcus* screening era. Early-onset sepsis was mainly caused by group B *Streptococcus*, *Streptococcus bovis* group, and *Escherichia coli.* In late-onset sepsis, coagulase-negative *Staphylococcus* and non-*Escherichia coli* Gram-negative bacteria and yeasts assumed significance. Overall, cefotaxime consumption had increased in the latter half of the study period. Within these five years when the COVID-19 pandemic reigned, there was a significant drop in neonatal sepsis, especially late-onset sepsis. There is a need for antimicrobial stewardship in the neonatal unit to optimise appropriate therapy for sick neonates, particularly in premature neonates where the concordance of empirical antimicrobial therapy was lower.

## Figures and Tables

**Table 1:** Demographics of neonates with neonatal sepsis in Tuen Mun Hospital, 2014–2023.

Period		2014–2018	2019–2023	OR [95% CI]	*p*-Value	Total
Number of Patients		89	29			118
Sex	Male	44 (49.4%)	15 (51.7%)	1.10 [0.44, 2.77]	1	59 (50.0%)
	Female	45 (50.6%)	14 (48.3%)			59 (50.0%)
Birth weight	V/ELBW (<1.5 kg)	26 (29.2%)	8 (27.6%)	0.92 [0.31, 2.52]	1	34 (28.8%)
	N/LBW (≥1.5 kg)	63 (70.8%)	21 (72.4%)			84 (71.2%)
Onset	EOS	21 (23.6%)	7 (24.1%)	1.03 [0.33, 2.96]	1	28 (23.7%)
	LOS	68 (76.4%)	22 (75.9%)			90 (76.3%)
	CA-LOS	17 (19.1%)	7 (24.1%)	1.4 [0.41, 4.43]	0.58	24 (20.4%)
	HA-LOS	51 (57.3%)	15 (51.7%)			66 (55.9%)
Hospitalisation	In-born	68 (76.4%)	21 (72.4%)	0.81 [0.29, 2.44]	0.8	89 (75.4%)
	Out-born	21 (23.6%)	8 (27.6%)			29 (24.6%)
Prior antibiotic in the intrapartum or neonatal period	Naive	32 (36.0%)	10 (34.5%)	0.94 [0.35, 2.43]	1	42 (35.6%)
	Exposed	57 (64.0%)	19 (65.5%)			76 (64.4%)

Abbreviations—OR: odds ratio, CI: confidence interval, V/ELBW: very low/extremely low birth weight, N/LBW: low/normal birth weight, EOS: early-onset sepsis, LOS: late-onset sepsis, CA-LOS: community-acquired LOS, HA-LOS: healthcare-associated LOS.

**Table 2:** Culture isolates of neonatal sepsis in Tuen Mun Hospital, 2014–2023, by period.

	2014–2018	2019–2023	OR [95% CI]	*p*-Value	Total
Culture isolates	94	31			125
Gram-positive bacteria	49 (52.1%)	13 (41.9%)	0.66 [0.27, 1.62]	0.41	62 (49.6%)
*Streptoccocus agalactiae*	13 (13.8%)	3 (9.7%)	0.67 [0.11, 2.70]	0.76	16 (12.8%)
*Streptococcus bovis* group	8 (8.5%)	0	-	0.2	8 (6.4%)
*Streptococcus mitis* group, nonpneumococcal	2 (2.1%)	0	-	1	2 (1.6%)
*Streptococcus pneumoniae*	1 (1.1%)	0	-	1	1 (0.8%)
Coagulase-negative *Staphylococcus*	20 (21.3%)	9 (29.0%)	1.51 [0.53, 4.10]	0.46	29 (23.2%)
*Staphylococcus aureus*	2 (2.1%)	0	-	1	2 (1.6%)
*Enterococcus faecalis*	3 (3.2%)	1 (3.3%)	1.01 [0.02, 13.1]	1	4 (3.2%)
Gram-negative bacteria	40 (42.6%)	17 (54.8%)	1.64 [0.67, 4.04]	0.3	57 (45.6)
*Escherichia coli*	22 (23.4%)	11 (35.5%)	1.8 [0.67, 4.67]	0.24	33 (26.4%)
*Klebsiella pneumoniae* complex	7 (7.4%)	2 (6.5%)	0.86 [0.08, 4.86]	1	9 (7.2%)
*Klebsiella aerogenes*	3 (3.2%)	0	-	0.57	3 (2.4%)
*Enterobacter cloacae* complex	3 (3.2%)	2 (6.5%)	2.09 [0.17, 19.1]	0.6	5 (4%)
*Morganella morganii*	0	1 (3.3%)	-	0.25	1 (0.8%)
*Salmonella* spp.	2 (2.2%)	0	-	1	2 (1.6%)
*Pseudomonas aeruginosa*	1 (1.1%)	0	-	1	1 (0.8%)
*Campylobacter jejuni*	1 (1.1%)	1 (3.3%)	3.1 [0.04, 246]	0.44	2 (1.6%)
*Neisseria meningitidis*	1 (1.1%)	0	-	1	1 (0.8%)
Yeasts	5 (5.3%)	1 (3.2%)	0.59 [0.01, 5.63]	1	6 (4.8%)
*Candida albicans*	3 (3.2%)	0	-	0.57	3 (2.4%)
*Candida tropicalis*	1 (1.1%)	0	-	1	1 (1.6%)
*Candida orthopsilosis*	0	1 (3.2%)	-	0.25	1 (1.6%)
*Lodderomyces elongisporus*	1 (1.1%)	0	-	1	1 (1.6%)

Abbreviations—OR: odds ratio, CI: confidence interval.

**Table 3:** Patient characteristics and culture isolates of neonatal sepsis in Tuen Mun Hospital, 2014–2023, by birth weight.

		V/ELBW (<1.5 kg)	N/LBW (≥1.5 kg)	OR [95% CI]	*p*-Value
Number of patients	Total	34	84		
Onset	EOS	5 (14.7%)	23 (27.4%)	0.46 [0.12, 1.41]	0.16
	LOS	29 (85.3%)	61 (72.6%)		
Hospitalisation	In-born	34 (100%)	55 (65.5%)	-	<0.001
	Out-born	0	29 (34.5%)		
Prior antibiotic in the intrapartum or neonatal period	Naive	3 (8.8%)	39 (46.4%)	0.11 [0.02, 0.41]	0.001
	Exposed	31 (91.2%)	45 (53.6%)		
Culture isolates	Total	38	87		
Gram-positive bacteria		21 (55.3%)	41 (47.1%)	1.39 [0.60, 3.21]	0.44
*Streptoccocus agalactiae*		3 (7.9%)	13 (14.9%)	0.49 [0.08, 1.95]	0.39
*Streptococcus bovis* group		1 (2.6%)	7 (8.0%)	0.31 [0.01, 2.57]	0.43
*Streptococcus mitis* group, nonpneumococcal		0	2 (2.3%)	-	1
*Streptococcus pneumoniae*		0	1 (1.1%)	-	1
Coagulase-negative *Staphylococcus*		15 (39.5%)	14 (16.1%)	3.40 [1.30, 8.83]	0.006
*Staphylococcus aureus*		1 (2.6%)	1 (1.1%)	2.32 [0.03, 185]	0.52
*Enterococcus faecalis*		1 (2.6%)	3 (3.4%)	0.76 [0.01, 9.81]	1
Gram-negative bacteria		11 (28.9%)	46 (52.9%)	0.36 [0.14, 0.88]	0.019
*Escherichia coli*		5 (13.6%)	28 (32.2%)	0.32 [0.09, 0.96]	0.029
*Klebsiella pneumoniae* complex		2 (5.3%)	7 (8.0%)	0.63 [0.06, 3.58]	0.72
*Klebsiella aerogenes*		1 (2.6%)	2 (2.3%)	1.15 [0.02, 22.7]	1
*Enterobacter cloacae* complex		2 (5.3%)	3 (3.4%)	1.56 [0.12, 14.1]	0.64
*Morganella morganii*		0	1 (1.1%)	-	1
*Salmonella* spp.		0	2 (2.3%)	-	1
*Pseudomonas aeruginosa*		1 (2.6%)	0	-	0.3
*Campylobacter jejuni*		0	2 (2.3%)	-	1
*Neisseria meningitidis*		0	1 (1.1%)	-	1
Yeasts		6 (15.8%)	0	-	<0.001

Abbreviations—OR: odds ratio, CI: confidence interval, V/ELBW: very low/extremely low birth weight, N/LBW: low/normal birth weight, EOS: early-onset sepsis, LOS: late-onset sepsis.

**Table 4:** Patient characteristics and culture isolates of neonatal sepsis in Tuen Mun Hospital, 2014–2023, by sepsis onset.

		EOS	LOS	OR [95% CI]	*p*-Value
Number of patients		28	90		
Birth weight	V/ELBW (<1.5 kg)	5 (17.8%)	29 (32.2%)	2.19 [0.71, 8.07]	0.16
	N/LBW (≥1.5 kg)	23 (82.1%)	61 (67.8%)		
Hospitalisation	In-born	28 (100%)	61 (67.8%)	-	<0.001
	Out-born	0	29 (32.2%)		
Prior antibiotic in the intrapartum or neonatal period	Naive	17 (60.7%)	25 (27.8%)	0.25 [0.09, 0.66]	0.003
	Exposed	11 (39.3%)	65 (72.2%)		
Culture isolates	Total	29	96		
Gram-positive bacteria		18 (62.1%)	44 (45.8%)	0.52 [0.20, 1.31]	0.14
*Streptoccocus agalactiae*		9 (32.1%)	7 (7.3%)	0.17 [0.05–0.61]	0.002
*Streptococcus bovis* group		7 (24.1%)	1 (1.0%)	0.03 [0.0007, 0.29]	<0.001
*Streptococcus mitis* group, nonpneumococcal		0	2 (2.1%)	-	1
*Streptococcus pneumoniae*		1 (3.4%)	0	-	0.23
Coagulase-negative *Staphylococcus*		1 (3.4%)	28 (31.1%)	11.5 [1.70, 488]	0.003
*Staphylococcus aureus*		0	2 (2.1%)	-	1
*Enterococcus faecalis*		0	4 (4.2%)	-	0.57
Gram-negative bacteria		11 (37.9%)	46 (47.9%)	1.51 [0.60, 3.92]	0.4
*Escherichia coli*		11 (37.9%)	22 (22.9%)	0.49 [0.18, 1.33]	0.15
Non-*Escherichia coli*		0	24 (25.0%)	-	0.001
*Klebsiella pneumoniae* complex		0	9 (9.4%)	-	0.12
*Klebsiella aerogenes*		0	3 (3.1%)	-	1
*Enterobacter cloacae* complex		0	5 (5.2%)	-	0.59
*Morganella morganii*		0	1 (1.0%)	-	1
*Salmonella* spp.		0	2 (2.1%)	-	1
*Pseudomonas aeruginosa*		0	1 (1.0%)	-	1
*Campylobacter jejuni*		0	2 (2.1%)	-	1
*Neisseria meningitidis*		0	1 (1.0%)	-	1
Yeasts		0	6 (6.3%)	-	0.33

Abbreviations—OR: odds ratio, CI: confidence interval, V/ELBW: very low/extremely low birth weight, N/LBW: low/normal birth weight, EOS: early-onset sepsis, LOS: late-onset sepsis.

**Table 5:** Patient characteristics and culture isolates of LOS in Tuen Mun Hospital, 2014–2023.

		CA-LOS	HA-LOS	OR [95% CI]	*p*-Value
Number of patients	Total	24	66		
Birth weight	V/ELBW (<1.5 kg)	0	29 (43.9%)	-	<0.001
	N/LBW (≥1.5 kg)	24 (100%)	37 (56.1%)		
Hospitalisation	In-born	0	60 (90.9%)	-	<0.001
	Out-born	24 (100%)	6 (9.1%)		
Prior antibiotic in the intrapartum or neonatal period	Naive	13 (54.2%)	12 (18.2%)	0.19 [0.06, 0.59]	0.001
	Exposed	11 (45.8%)	54 (81.8%)		
Culture isolates	Total	24	72		
Gram-positive bacteria		11 (45.8%)	33 (45.8%)	1.0 [0.36, 2.83]	1
*Streptoccocus agalactiae*		7 (29.2%)	0	-	<0.001
*Streptococcus bovis* group		0	1 (1.4%)	-	1
*Streptococcus mitis* group, nonpneumococcal		1 (4.2%)	1 (1.4%)	0.32 [0.004, 26.5]	0.44
*Streptococcus pneumoniae*		0	0	-	-
Coagulase-negative *Staphylococcus*		3 (12.5%)	25 (34.7%)	3.72 [0.96, 21.2]	0.042
*Staphylococcus aureus*		0	2 (2.8%)	-	1
*Enterococcus faecalis*		0	4 (5.6%)	-	0.57
Gram-negative bacteria		13 (54.2%)	33 (54.2%)	0.72 [0.25, 2.00]	0.49
*Escherichia coli*		8 (33.3%)	14 (19.4%)	0.48 [0.16, 1.59]	0.17
*Klebsiella pneumoniae* complex		0	9 (12.5%)	-	0.11
*Klebsiella aerogenes*		0	3 (4.2%)	-	0.57
*Enterobacter cloacae* complex		1 (4.2%)	4 (5.6%)	1.35 [0.12, 69.5]	1
*Morganella morganii*		0	1 (1.4%)	-	1
*Salmonella* spp.		1 (4.2%)	1 (1.4%)	0.32 [0.004, 26.5]	0.44
*Pseudomonas aeruginosa*		0	1 (1.4%)	1	1
*Campylobacter jejuni*		2 (8.3%)	0	-	0.06
*Neisseria meningitidis*		1 (4.2%)	0	-	0.25
Yeasts		0	6 (8.3%)	-	0.33

Abbreviations—OR: odds ratio, CI: confidence interval, V/ELBW: very low/extremely low birth weight, N/LBW: low/normal birth weight, LOS: late-onset sepsis, CA-LOS: community-acquired LOS, HA-LOS: healthcare-associated LOS.

**Table 6:** Mortality cases of neonatal sepsis in Tuen Mun Hospital, 2014–2023.

Patient	Sex	Gestation (Weeks)	Birth Weight (kg)	Age at Which Positive Culture Obtained (Days)	Specimen	Organism	Empirical Antimicrobial(s) Given	Concordance
1	F	25	0.750	0	Blood	*Streptococcus agalactiae*	Ampicillin, gentamicin	+
2	F	25	0.665	0	Blood	*Escherichia coli*	Died before antimicrobial commenced	N/A
3	F	33	1.520	9	Blood	*Escherichia coli*	Cefotaxime, vancomycin, metronidazole	+
4	M	24	0.625	6	Blood	*Pseudomonas aeruginosa*	Meropenem, vancomycin	+
5	M	23	0.520	11	Blood	*Candida albicans*	Cefotaxime, teicoplanin	−
6	M	24	0.690	13	Blood	*Candida albicans*	Meropenem, vancomycin	−
7	M	23	0.670	3	Blood	*Candida tropicalis*	Cefotaxime, vancomycin	−
8	M	24	0.740	11	Blood	*Lodderomyces elongisporus*	Cefotaxime, vancomycin, metronidazole	−

Abbreviations—M: male, F: female; +: concordant, −: discordant, N/A: not applicable.

**Table 7:** Nonsusceptibility of isolates* found in neonatal sepsis in Tuen Mun Hospital, 2014–2023.

		Period				Birth Weight				Onset				
Organisms	Antibiotic	2014–2018	2019–2023	OR [95% CI]	*p*-Value	N/LBW (≥1.5 kg)	V/ELBW (<1.5 kg)	OR [95% CI]	*p*-Value	EOS	LOS		OR [95% CI]	*p*-Value
											CA-LOS	HA-LOS		
Staphylococci	Methicillin	18/22 (81.8%)	7/9 (77.8%)	0.78 [0.09, 10.5]	1.0	9/15 (60.0%)	16/16 (100%)	-	0.006	0/1	25/30 (83.3%)		-	0.19
											0/3	25/27 (92.6%)	-	0.003
Streptococci	Penicillin	2/24 (8.3%)	0/3	-	1.0	2/23 (8.7%)	0/4	-	1.0	0/17	2/10 (20%)		-	0.13
											1/8 (12.5%)	1/2 (50.0%)	-	0.38
Gram-negative bacteria	Ampicillin	25/28 (89.2%)	13/15 (86.7%)	0.78 [0.12, 5.27]	1.0	30/35 (85.7%)	8/8 (100%)	-	0.56	8/9 (88.9%)	30/34 (88.2%)		7.0 [0.04, 626]	1.0
											7/9 (77.8%)	23/25 (92.0%)	0.94 [0.02, 11.4]	0.28
	Cefotaxime/ceftriaxone	5/38 (13.2%)	4/16 (25.0%)	2.2 [0.37, 12.1]	0.42	4/43 (9.3%)	5/11 (45.5%)	8.13 [1.27, 51.8]	0.01	0/11	9/43 (20.9%)		3.29 [0.20, 51.2]	0.18
											1/11 (9.1%)	8/32 (25.0%)	-	0.41
	Gentamicin	4/36 (11.1%)	4/16 (25.0%)	2.67 [0.42, 16.5]	0.23	7/41 (17.1%)	1/11 (9.1%)	0.49 [0.01, 4.65]	1.0	4/11 (36.4%)	4/41 (9.6%)		3.33 [0.35, 162]	0.052
											0/9	4/34 (11.8%)	0.19 [0.03, 1.32]	1.0

* Where tested or able to be inferred. Abbreviations—OR: odds ratio, CI: confidence interval, V/ELBW: very low/extremely low birth weight, N/LBW: low/normal birth weight, EOS: early-onset sepsis, LOS: late-onset sepsis, CA-LOS: community-acquired LOS, HA-LOS: healthcare-associated LOS.

**Table 8:** Patterns of empirical antibiotic prescription by frequency for neonatal sepsis in Tuen Mun Hospital, 2014–2023.

	Period				Birth Weight				Onset				
Antimicrobial	2014–2018	2019–2023	OR [95% CI]	*p*-value	N/LBW (≥1.5 kg)	V/ELBW (<1.5 kg)	OR [95% CI]	*p*-Value	EOS	LOS		OR [95% CI]	*p*-Value
										CA-LOS	HA-LOS		
Penicillin	2 (2.2%)	0	-	1.0	1 (1.2%)	1 (2.9%)	2.52 [0.03, 200]	0.495	2 (7.1%)	0		-	0.055
Ampicillin	55 (61.8%)	19 (65.5%)	1.17 [0.45, 3.18]	0.83	68 (81.0%)	6 (17.6%)	0.05 [0.02, 0.15]	<0.001	25 (89.3%)	49 (54.4%)		0.14 [0.03, 0.53]	<0.001
										24 (100%)	25 (37.9%)	-	<0.001 ^#^
Cefotaxime	58 (65.2%)	28 (96.6%)	15.0 [2.21, 631]	<0.001	59 (70.2%)	27 (79.4%)	1.63 [0.59, 5.02]	0.37	9 (32.1%)	77 (85.6%)		12.5 [4.21, 38.0]	<0.001
										22 (91.6%)	55 (83.3%)	0.45 [0.05, 2.36]	0.50 ^#^
Meropenem	4 (4.5%)	0	-	0.57	1 (1.2%)	3 (8.8%)	8.03 [0.61, 427]	0.07	0	4 (4.4%)		-	0.57
										0	4 (6.1%)	-	0.57 ^#^
Gentamicin	24 (27.0%)	3 (10.3%)	0.31 [0.06, 1.18]	0.08	22 (26.2%)	5 (14.7%)	0.49 [0.13, 1.50]	0.23	18 (64.3%)	9 (10.0%)		0.06 [0.02, 0.19]	<0.001
										2 (8.3%)	7 (10.6%)	1.31 [0.22, 13.8]	1.0 ^#^
Vancomycin	23 (25.8%)	10 (34.5%)	1.51 [0.54, 4.02]	0.48	12 (14.3%)	21 (61.7%)	9.69 [3.51, 27.1]	<0.001	0	33 (36.7%)		-	<0.001
										0	33 (50.0%)	-	<0.001 ^#^
Teicoplanin	1 (1.1%)	0	-	1.0	0	1 (2.9%)	-	0.29	0	1 (1.1%)		-	1.0
										0	1 (1.5%)	-	1.0 ^#^
Metronidazole	7 (7.9%)	3 (10.3%)	1.35 [0.21, 6.45]	0.71	9 (10.7%)	1 (2.9%)	0.25 [0.01, 1.97]	0.28	2 (7.1%)	8 (8.9%)		1.27 [0.23, 13.0]	1.0
										0	8 (12.1%)	-	0.10 ^#^
Amphotericin B	1 (1.1%)	0	-	1.0	0	1 (2.9%)	-	0.29	0	1 (1.1%)		-	1.0
										0	1 (1.5%)	-	1.0 ^#^

#—Comparing CA-LOS versus HA-LOS. Abbreviations—OR: odds ratio, CI: confidence interval, V/ELBW: very low/extremely low birth weight, N/LBW: low/normal birth weight, EOS: early-onset sepsis, LOS: late-onset sepsis, CA-LOS: community-acquired LOS, HA-LOS: healthcare-associated LOS.

**Table 9:** Concordance of empiric antimicrobial therapy in neonatal sepsis in Tuen Mun Hospital, 2014–2023.

	2014–2018	2019–2023	OR [95% CI]	*p*-Value	N/LBW (≥1.5 kg)	V/ELBW (<1.5 kg)	OR [95% CI]	*p*-Value	EOS	LOS		OR [95% CI]	*p*-Value
										CA-LOS	HA-LOS		
All antimicrobial prescriptions	70/86 (81.4%)	19/28 (67.9%)	0.48 [0.17, 1.45]	0.19	69/81 (85.2%)	20/33 (60.6%)	0.27 [0.10, 0.76]	0.006	25/27 (92.6%)	64/87 (73.6%)		0.22 [0.02, 1.03]	0.06
										20/22 (90.9%)	44/65 (67.7%)	0.40 [0.18, 0.88]	0.048
Empirical prescriptions that included:													
Ampicillin and gentamicin	18/20 (90.0%)	1/3 (33.3%)	0.06 [0.0009, 1.84]	0.07	17/21 (81.0%)	2/2 (100%)	-	1.0	14/16 (87.5%)	5/7 (71.4%)		0.36 [0.02, 6.47]	0.56
										1/2 (50.0%)	4/5 (80.0%)	4.0 [0.03, 391]	1.0
Ampicillin and cefotaxime	31/34 (91.2%)	12/15 (80.0%)	0.39 [0.05, 3.37]	0.35	41/45 (91.1%)	2/4 (50.0%)	0.10 [0.01, 1.83]	0.07	9/9 (100%)	34/40 (85.0%)		-	0.58
										19/20 (95.0%)	15/20 (75.0%)	0.16 [0.003, 1.70]	0.18
Vancomycin and cefotaxime	13/20 (65.0%)	5/9 (55.6%)	0.67 [0.10, 4.64]	0.69	7/11 (63.6%)	11/18 (61.1%)	0.90 [0.14, 5.35]	1.0	0	0	18/29 (62.1%)	-	-
Other antibiotics	8/12 (66.7%)	1/1 (100%)	-	1.0	4/4 (100%)	5/9 (55.6%)	-	0.23	2/2 (100%)	0	7/11 (63.6%)	-	1.0

Abbreviations—OR: odds ratio, CI: confidence interval, V/ELBW: very low/extremely low birth weight, N/LBW: low/normal birth weight, EOS: early-onset sepsis, LOS: late-onset sepsis, CA-LOS: community-acquired LOS, HA-LOS: healthcare-associated LOS.

## Data Availability

The original contributions presented in this study are included in the article material. Further enquiries can be directed to the corresponding author.

## Abbreviations

The following abbreviations are used in this manuscript:

EOS	Early-onset sepsis
LOS	Late-onset sepsis
CA-LOS	Community-acquired late-onset sepsis
HA-LOS	Healthcare-associated late-onset sepsis
V/ELBW	Very low/extremely low birth weight
N/LBW	Normal/low birth weight
GBS	*Streptococcus agalactiae* (group B *Streptococcus*)
SBG	*Streptococcus bovis* group
CoNS	Coagulase-negative *Staphylococcus*
OR	Odds ratio
CI	Confidence interval
